# Comment on “evaluation of the prevalence of overactive bladder syndrome in patients with rheumatoid arthritis”

**DOI:** 10.1186/s42358-026-00558-8

**Published:** 2026-06-16

**Authors:** Qodirova Marhabo, Mamatkhujaeva Gulnarahan, Sakhatalieva Raykhana, Boboyorov Rasul, Arman Abroumand Gholami

**Affiliations:** 1 Department of Propedeutics of Children’s Diseases, Samarkand State Medical University, Samarkand, Uzbekistan; 2 Department of Ophthalmology, Otorhinolaryngology, Oncology, Urology, and Endourology, Faculty of Advanced Training and Retraining of Physicians, Andijan State Medical Institute, Andijan, Uzbekistan; 3 Department of Urology, Andijan State Medical Institute, Andijan, Uzbekistan; 4 Department of Otorhinolaryngology, Pediatric Otorhinolaryngology, Tashkent State Medical University, Tashkent, Uzbekistan; 5 Nervous System Stem Cell Research Center, Semnan University of Medical Sciences, Semnan, Iran; 6 Department of Anatomy and Cell Biology, School of Medicine, Mashhad University of Medical Sciences, Mashhad, Iran; 7 Neuroscience Research Center, Mashhad University of Medical Sciences, Mashhad, Iran

**Keywords:** Rheumatoid arthritis, Overactive bladder, Central sensitization, Logistic regression, Multicollinearity, Dichotomisation

## Abstract

We read with interest the study by Cirakoglu and Yuce on overactive bladder (OAB) and central sensitization (CS) in rheumatoid arthritis (RA) patients. While the study provides valuable hypothesis‑generating insights, several methodological limitations warrant consideration. The OAB-V8 cut‑off of 8 was validated in a primary care population, not in RA patients, and dichotomising this ordinal scale discards information and reduces statistical power. The regression model included correlated variables (HAQ-DI, VAS-pain, DAS-28) without testing for multicollinearity, which can inflate standard errors and produce unreliable p‑values. Additionally, the authors did not verify key logistic regression assumptions, including linearity of continuous variables with the logit and absence of influential outliers. These limitations suggest that the findings should be interpreted as hypothesis‑generating rather than definitive. Future studies should address these methodological issues to clarify the relationship between CS and OAB in RA.

**Dear Editor**

We read with great interest the article by Cirakoglu and Yuce entitled “Evaluation of the prevalence of overactive bladder syndrome in patients with rheumatoid arthritis” [1]. The study provides important insights into the association between overactive bladder (OAB) and central sensitization (CS) in rheumatoid arthritis (RA) patients. However, several methodological issues warrant discussion as they may influence the interpretation of the findings.

First, the authors used an OAB-V8 cut‑off of 8 based on validation in a primary care population [2]. However, this cut‑off has not been validated in patients with RA, a population with distinct symptom profiles, medication regimens, and inflammatory burden. The OAB-V8 is a screening tool, not a diagnostic instrument, and its performance characteristics may differ substantially in RA patients. Moreover, dichotomising an ordinal scale discards information, reduces statistical power, and may increase the risk of false‑positive findings [3]. The authors themselves reported a borderline p‑value for age (*p* = 0.054), and the OR for CS is very close to 1.0 (1.048). The additional loss of power from dichotomisation may have further compromised the study’s ability to detect true associations. An ordinal logistic regression would have preserved the full score range and could have revealed whether the association with CS is consistent across the entire symptom severity spectrum.

Second, the regression model included HAQ-DI, VAS-pain, and DAS-28 simultaneously. These variables are conceptually and statistically correlated. Indeed, a study in RA patients demonstrated that the effect of DAS-28 on functional disability overlaps substantially with the effect of pain VAS [4]. Entering all three together without testing for multicollinearity is problematic. Multicollinearity inflates standard errors, produces unreliable p‑values and confidence intervals, and can make individual predictors appear non‑significant while the overall model remains significant [5]. The authors did not report variance inflation factor or correlation matrices, leaving the extent of multicollinearity unexamined and the stability of the reported coefficient estimates uncertain.

Lastly, logistic regression relies on several assumptions that the authors did not verify. The linearity assumption requires that continuous independent variables have a linear relationship with the logit of OAB. Unassessed non‑linearity can lead to model misspecification and biased coefficient estimates. Additionally, the authors did not report diagnostic checks for influential outliers or for complete or quasi‑complete separation. These omissions leave the robustness of the reported associations uncertain.

In conclusion, the study provides a valuable hypothesis‑generating contribution to the understanding of OAB and CS in RA patients. However, the methodological limitations outlined above, unvalidated cut‑off, dichotomisation of an ordinal scale, unexamined multicollinearity, and unverified regression assumptions, warrant caution in overinterpreting the findings as definitive evidence. Future studies should address these methodological issues to clarify the true relationship between CS and OAB in RA.

## Data Availability

No datasets were generated or analysed during the current study.

## Abbreviations

OAB: Overactive bladder
CS: Central sensitization
RA: Rheumatoid arthritis
VAS: Visual Analog Scale
HAQ-DI: Health Assessment Questionnaire Disability Index
DAS-28: Disease Activity Score in 28 joints
OAB-V8: Overactive Bladder Questionnaire – Version 8
OR: Odds ratio
CI: Confidence interval
